# The disengagement of visual attention in the gap paradigm across adolescence

**DOI:** 10.1007/s00221-017-5085-2

**Published:** 2017-09-07

**Authors:** S. Van der Stigchel, R. S. Hessels, J. C. van Elst, C. Kemner

**Affiliations:** 1 Department of Experimental Psychology, Helmholtz Institute, Utrecht University, Heidelberglaan 1, 3584 CS Utrecht, The Netherlands; 2 Developmental Psychology, Utrecht University, Utrecht, The Netherlands; 3 Brain Centre Rudolf Magnus, University Medical Center, Utrecht, The Netherlands

**Keywords:** Gap effect, Eye movements, Development, Attentional disengagement

## Abstract

Attentional disengagement is important for successful interaction with our environment. The efficiency of attentional disengagement is commonly assessed using the gap paradigm. There is, however, a sharp contrast between the number of studies applying the gap paradigm to clinical populations and the knowledge about the underlying developmental trajectory of the gap effect. The aim of the present study was, therefore, to investigate attentional disengagement in a group of children aged 9–15. Besides the typically deployed gap and the overlap conditions, we also added a baseline condition in which the fixation point was removed at the moment that the target appeared. This allowed us to reveal the appropriate experimental conditions to unravel possible developmental differences. Correlational analyses showed that the size of the gap effect became smaller with increasing age, but only for the difference between the gap and the overlap conditions. This shows that there is a gradual increase in the capacity to disengage visual attention with increasing age, but that this effect only becomes apparent when the gap and the overlap conditions are compared. The gradual decrease of the gap effect with increasing age provides additional evidence that the attentional system becomes more efficient with increasing age and that this is a gradual process.

## Introduction

Because we can only execute one eye movement at a time, there is a continuous decision process to determine to which location the next eye movement will be executed. Research has shown that there are different stages of this decision process that show a developmental trajectory. In the present study, we will focus on the disengagement of visual attention. Because of the obligatory link between attention and eye movements (Rizzolatti et al. 1994), the disengagement of attention from the current fixation location is necessary in order to program the next saccade. The efficiency of this disengagement can be measured using the gap paradigm, in which participants have the task to execute a saccade from a central fixation point to a peripheral target. By removing the fixation point before the onset of the target, the participant can already disengage from the fixation point before the target appears. This early disengagement results in a reduction in saccade latency compared to when the fixation point remains present (the so called “gap effect”, Saslow 1967). This robust reduction in latency is independent of advance knowledge of the location of the saccade target (Walker et al. 1995).

There is now ample evidence that the size of the gap effect is larger for children compared to adults (Cohen and Ross 1977, 1978; Klein 2001), meaning that it takes children longer to disengage attention than adults. This result shows that the attentional system becomes more efficient during childhood. Attentional disengagement is important for successful interaction with our environment, such as being safe in traffic or during child–parent interactions. Because of the importance of attentional disengagement, the gap effect has been studied in various clinical populations, such as children with autism spectrum disorder (Fischer et al. 2014; Goldberg et al. 2002; Landry and Bryson 2004; van der Geest et al. 2001) and children with ADHD (Cairney et al. 2001; Munoz et al. 2003). Especially for studies on children with autism spectrum disorder, the results have been inconclusive, with some studies showing a smaller gap effect (van der Geest et al. 2001), other studies reporting a larger gap effect (Landry and Bryson 2004), and other studies showing no modulation of the gap effect in children with autism spectrum disorder compared to controls (Goldberg et al. 2002).

The inconsistent clinical results discussed above emphasize the need for more knowledge about the mechanisms underlying the gap effect. There is namely a sharp contrast between the number of studies applying the gap paradigm to clinical populations and the knowledge about the developmental trajectory of the gap effect. Although the differences between children and adults in the size of the gap effect are well established (Cohen and Ross 1977, 1978; Klein 2001), findings regarding the exact developmental trajectory of the gap effect during development have been largely inconsistent. Whereas some studies observed no correlation between age and the gap effect in participants ranging between 7 and 12 years (Eenshuistra et al. 2007; Goepel et al. 2011); Klein (2001; Klein and Foerster 2001) showed that the size of the gap effect decreased with age within their group of children ranging between 6 and 11 years. As knowledge about the exact developmental differences in the gap effect is important for the correct interpretation of clinical studies, the first aim of the present study was to investigate whether the size of the gap effect differs between 9-, 12-, and 15-year-old children using a relatively large sample size.

Developmental differences of the gap effect might be especially interesting given the dissociation in the maturation of the different neural areas involved in the gap effect. It is known that the offset of the fixation point reduces activation at both the fixation location in the saccade map in the subcortical superior colliculus (Dorris and Munoz 1995), and in the frontal eye fields in the frontal cortex (Dias and Bruce 1994). Whereas frontal cortical regions are characterized by considerable developmental changes (Fuster 1997), subcortical areas, such as the superior colliculus, are already matured in the infant (Stampalija and Kostovic 1981; Yakovlev and Lecours 1967). A gradual decrease of the gap effect with increasing age would, therefore, be consistent with the idea that the gap effect can be adopted as a measure of frontal cortex development (e.g., Munoz et al. 2003).

A second aim of this study was to investigate the appropriate task parameters to reveal the possible developmental trajectory of the gap effect. Infant studies on the gap effect often include a baseline (or ‘no gap’) condition in which the onset of the peripheral stimulus immediately follows the offset of the central stimulus (e.g., Hood and Atkinson 1993; Nakagawa and Sukigara 2013). This condition is generally not included in studies in older children, adolescents, and adults, based on the finding that the processes thought to be triggered by the fixation point offset need about 200 ms to fully develop (Klein and Foerster 2001; Reuter-Lorenz et al. 1991). Therefore, to measure the disengagement process to its full extent, it is argued that one needs to compare behavior in a condition in which the disengagement process is complete to a condition in which there is little to no disengagement (see, e.g., Cousijn et al. 2017). In the gap condition, the disengagement is complete, whereas there is little to no disengagement in the overlap condition. The baseline condition reflects an intermediate condition, because disengagement is already partly facilitated by the removal of the fixation stimulus. To investigate the assumption that the overlap condition is more appropriate to assess the gap effect than the baseline condition, we specifically compared the gap effect as measured by the difference between the gap and overlap conditions and the gap effect as measured by the difference between the gap and baseline conditions. This comparison will reveal whether the possible developmental differences are indeed most pronounced in the difference between the gap and the overlap conditions.

## Methods

### Participants

Children from three different age groups were invited into the laboratory center: 9-year- (*n* = 36), 12-year- (*n* = 34), and 15-year- (*n* = 36) old healthy children with normal hearing and vision. Mean age of the three age groups was 8.9 (SD = 0.83), 11.9 (SD = 0.85), and 15.0 (SD = 0.84) years, respectively. There were 19 girls in the 9-year-old group, 16 girls in the 12-year-old group, and 21 girls in the 15-year-old group. Participants were recruited via the local municipality. This study was embedded in a larger project on the development of cognition at Utrecht University. The primary care-giver signed informed consent and received 10 Euro for each test-session. The project was approved by the Ethics Committee of the Faculty of Social and Behavioral Sciences, Utrecht University (protocol ID: FETC15-036).

### Gap-overlap task

The Gap-overlap task was based on Elsabbagh et al. (2013). Trials started with a clock (2.6° × 2.6°) expanding and contracting (maximum size 3.5° × 3.5°) at the center of the screen to attract the participant’s attention. After the participant fixated the central stimulus, it started spinning with a speed of 500°/s to maintain the participant’s attention. After 600–700 ms, a peripheral stimulus (a yellow oval, 2.6° × 2.6°) was presented at 19° to the left or right from the central stimulus. This 100 ms jitter in onset of the peripheral stimulus was implemented to decrease anticipatory saccades. The task contained a gap, overlap, and baseline condition. In the gap condition, central stimulus offset was 200.2 ms on average (SD = 1.69 ms, range 196.67–216.67 ms) before peripheral stimulus onset. For one participant, central stimulus offset was erroneously around 80 ms before peripheral target onset, and this participant was, therefore, excluded from the analyses. In the overlap condition, the central and peripheral stimulus remained simultaneously and inanimately on screen. In the baseline condition, the peripheral stimulus onset was at the same time as central stimulus offset. The peripheral stimulus stayed on screen until the participant fixated it or until 1500 ms elapsed. Upon fixating the peripheral stimulus, or if 1500 ms elapsed, the peripheral stimulus spun and contracted over 1000 ms. This feedback was combined with various sounds (e.g., a car horn, a bell). The Gap-overlap task consisted of 12 trials per condition, randomly presented. This relatively low number of trials was due to the time availability of the participants. It has previously been shown that a similar set-up provides sufficient statistical power to observe reliable differences between the gap, overlap, and the baseline condition (Cousijn et al. 2017).

### Apparatus

A Tobii TX300 eye-tracker (Tobii Technology, Stockholm, Sweden) with an integrated 23-inch monitor (1920 by 1080 pixels; 60 Hz refresh rate) was used to record eye movements. The Tobii TX300 ran at 300 Hz and communicated with MATLAB (version R2013a, MathWorks Inc., USA) and the Psych-Toolbox (version 3.0.11, Brainard 1997) running on a MacBook Pro (OS X 10.9) via the Tobii SDK.

### Procedure

Participants were positioned at a distance of 65 cm from the eye-tracker. A chin-rest was used to ensure the same distance and position throughout the experiment. Hereafter, an operator-controlled calibration was run, which consisted of colored expanding and contracting spirals presented at the four corners and the center of the screen. The spirals were accompanied by a sound. A web-cam was used to monitor the participant. When the operator judged the participant to be looking at the spiral, a button was pressed, after which the spiral contracted and was calibrated. Details of the calibration stimuli are given in Hessels et al. (2015). The operator judged the calibration output from the Tobii SDK, after which a decision was made to accept the calibration or re-calibrate.

After the calibration was accepted, the gap-overlap task was started. Throughout the experiment, the participant was monitored through the web-cam. The task including calibration lasted approximately 10–15 min.

### Data preparation and analyses

Eye-tracking data were pre-processed as follows: First, as the eye-tracking data sometimes contained extremely distant gaze coordinates, coordinates more than one screen distance outside the monitor were set to missing data. Hereafter, eye-tracking data containing validity codes higher than 1 were set to missing also (Niehorster et al. 2017). Validity codes over 1 indicate that the eye tracker was not sure from which eye it was recording, and may, therefore, report wrong gaze coordinates.

Because noise (i.e. low precision) may be a problem in developmental eye tracking (e.g., Hessels et al. 2015), which could lead to bursts of noise being labeled as saccades, saccadic reaction times (SRTs) were determined indirectly. First, fixations were identified in the eye-tracking data using the noise-robust Identification by 2-means clustering (I2MC) algorithm (Hessels et al. 2016b). As fixation offset corresponds to saccade onset, this approach allows accurate saccade onset labeling regardless of noise level (within a maximum of 2° of RMS noise). As the default settings of the I2MC may overestimate fixation duration when large saccades intersperse fixations (and subsequently overestimate SRT in the gap-overlap paradigm), one modification to the I2MC was made. Fixation start- and endpoints were determined by using the mean clustering weight signal plus 2 standard deviations (default parameters), and subsequently shifting backwards (for fixation offset) by 5 samples and forward (for fixation onset) by 6 samples. All other parameters were set to the default.

Following fixation detection, fixations were assigned to one of three areas of interest (AOIs): central stimulus, left peripheral stimulus, and right peripheral stimulus. This was done if at least 25% of the trial contained eye-tracking data. As previous research indicates that for sparse stimuli (i.e. stimuli containing few elements) outcome measures from large AOIs are most noise-robust (Hessels et al. 2016a), AOIs were the size of one-third of the screen. The left third belonged to the left peripheral stimulus, the middle third to the central stimulus, and the right third to the right peripheral stimulus. If the position of a fixation was inside one of these AOIs, it was assigned to this AOI, barring the following exception if the eye-tracking data which directly preceded the fixation contained more than 35% data loss, or contained a sample with a velocity of over 800°/s. In these cases, fixation position may be unreliable.

SRT was finally determined as the time between the offset of the last fixation on the central stimulus and the onset of the peripheral stimulus. If a fixation was made on the wrong side of the screen, or no fixation was made on the central stimulus, the SRT was excluded.

Because of the low number of trials, we computed the median SRT for each participant. We subsequently performed a repeated measures ANOVA with condition (gap, overlap, baseline) as within subject factor and group (9-, 12-, and 15-year-old) as a between subject factor. Post-hoc *t* tests were performed to explore possible main effects. Because we were specifically interested in the appropriate task parameters of the gap effect, we subsequently computed two difference scores. First, the gap-baseline effect was computed by subtracting the median SRTs of the gap condition from the median of the baseline condition for each participant. Second, the gap-overlap effect was computed by subtracting the median SRTs of the gap condition from the median of the overlap condition for each participant.

To examine how the gap-overlap and gap-baseline effect develop over age, we performed correlation analyses (Pearson) for both difference scores and the age of the participants. For this analysis, we adopted the actual age of the participants (in months), ranging between 99 and 203 months, instead of the categorization in three different groups. Pearson and Filon’s test for overlapping correlations for dependent groups (Diedenhofen and Musch 2015; Pearson and Filon 1898) was used to test whether these two correlations were significantly different.

## Results

### Data quality

Given the recent emphasis on reporting eye-tracking data quality in order to ensure the validity of eye-movement research (Holmqvist et al. 2012), measures for the accuracy and the precision of the eye-tracking signal are reported here. Precision was estimated by computing the average RMS noise across all fixations as detected by the I2MC algorithm. This gives an indication of the noise in the eye-tracking signal. Mean RMS noise of all fixations was 0.27° (SD = 0.83°) for the 9-year-old group, 0.36° (SD = 1.12°) for the 12-year-old group, and 0.25° (SD = 0.58°) for the 15-year-old group. Accuracy was estimated by taking the closest fixation to the central stimulus each trial, and computing the offset between its location and the center of the central stimulus. Fixations needed to be within 3.5° of the center of the central stimulus to be considered. The offset gives an indication of the discrepancy between gaze position as reported by the eye tracker and the assumed gaze position of the participant. Mean offset was 0.90° (SD = 0.51°) for the 9-year-old group, 0.86° (SD = 1.15°) for the 12-year-old group, and 0.88° (SD = 0.54°) for the 15-year-old group. Accuracy and precision for the Tobii TX300 under optimal conditions are 0.4° accuracy and 0.14° precision. While the achieved values for accuracy and precision are somewhat higher, they are well within limits for fixation detection (Hessels et al. 2016b) and AOI analysis (Hessels et al. 2016a).

Participants with less than eight identified SRTs per condition were excluded. Including the one participant that was excluded due to technical problems explained above, this criterion led to the exclusion of four participants in the 9-year-old group, three in the 12-year-old group and six in the 15-year-old group. The remaining number of participants was 32, 31, and 30, respectively.

### Saccadic reaction times

**Fig. 1:**
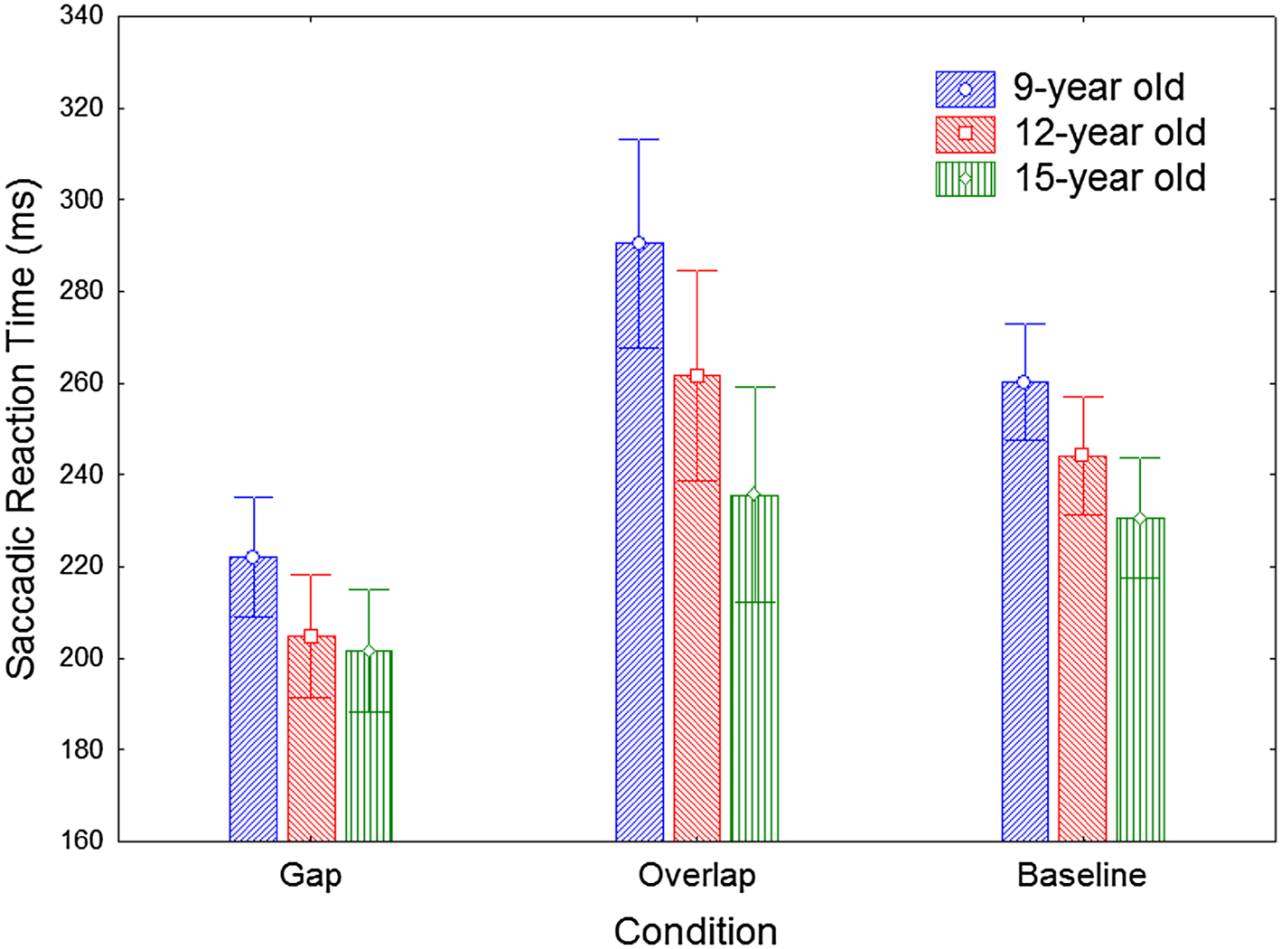
Result for the three conditions for the three different groups, showing the main effect of group (the 9-year-old group had longer SRTs than the 12-year-old group), the main effect of condition (overlap resulting in longer SRTs than baseline and gap conditions, with the gap condition resulting in shorter SRTs than the baseline condition), and the marginally significant interaction between condition and group (graphically illustrated in Fig. 2). Error bars indicate 95% CI

A repeated measures ANOVA with condition (gap, overlap, baseline) as within subject factor and group (9-, 12-, and 15-year-old) as a between-subject factor revealed a main effect of group [*F*(2,90) = 6.38; *p* < 0.01], a main effect of condition [*F*(2,180) = 57.76; *p* < 0.0001], and a marginally significant interaction between group and condition [*F*(4,180) = 2.18; *p* = 0.073]. Figure 1 shows the results for our main analysis (see also Table 1).

**Table 1:** Standard deviations and average number of included trials for all conditions

Group	Condition	Standard deviation of SRT (ms)	Average number of included trials
9-year-old	Gap	44	9.97
	Overlap	77	10.94
	Baseline	34	11.12
12-year-old	Gap	34	10.00
	Overlap	60	10.48
	Baseline	40	10.80
15-year-old	Gap	31	10.17
	Overlap	52	11.03
	Baseline	34	11.23

#### Main effect of group

The 9-year-old group showed longer SRTs (mean = 258 ms; SD = 42 ms) than the 12-year-old group [mean = 237 ms; SD = 39 ms; *t*(61) = 2.03; *p* < 0.05]. The 15-year-old group (mean = 223 ms; SD = 35 ms) did not differ significantly from the 12-year-old group [*t*(59) = 1.50; *p* = 0.14].

#### Main effect of condition

The three different conditions differed significantly. The gap condition (mean = 210 ms; SD = 38 ms) resulted in the shortest SRTs and differed significantly from the baseline condition [mean = 245 ms; SD = 37 ms; *t*(92) = 10.32, *p* < 0.0001] and the overlap condition [mean = 263 ms; SD = 68 ms; *t*(92) = 8.48, *p* < 0.0001]. The overlap condition had the longest SRTs [compared to the baseline condition *t*(92) = 3.50, *p* < 0.001].

#### (Marginal) interaction between group and condition

We investigated the interaction between group and condition by computing the gap-baseline and the gap-overlap effect.

For the gap-baseline effect, there were no significant differences between the groups (*p*’s > 0.17).

For the gap-overlap effect, there was a significant difference between the 9- and 15-year-old group [*t*(60) = 2.16; *p* < 0.04], caused by a larger overlap effect in the 9-year-old group (mean = 68 ms; SD = 78 ms) compared to the 15-year-old group (mean = 34 ms; SD = 40 ms).

The 9-year-old group did not differ from the 12-year-old group [mean = 57 ms; SD = 53 ms; *t*(61) = 0.68; *p* = 0.50]. There was a marginally significant difference between the 12-year- and the 15-year-old group [*t*(59) = 1.88; *p* = 0.07].

Although never used in the literature, we did compute the baseline-overlap effect for the sake of completeness. For this measure, there were no significant differences between the groups (*p*’s > 0.06).

#### Correlation between age and both the gap-overlap and the gap-baseline effect

**Fig. 2:**
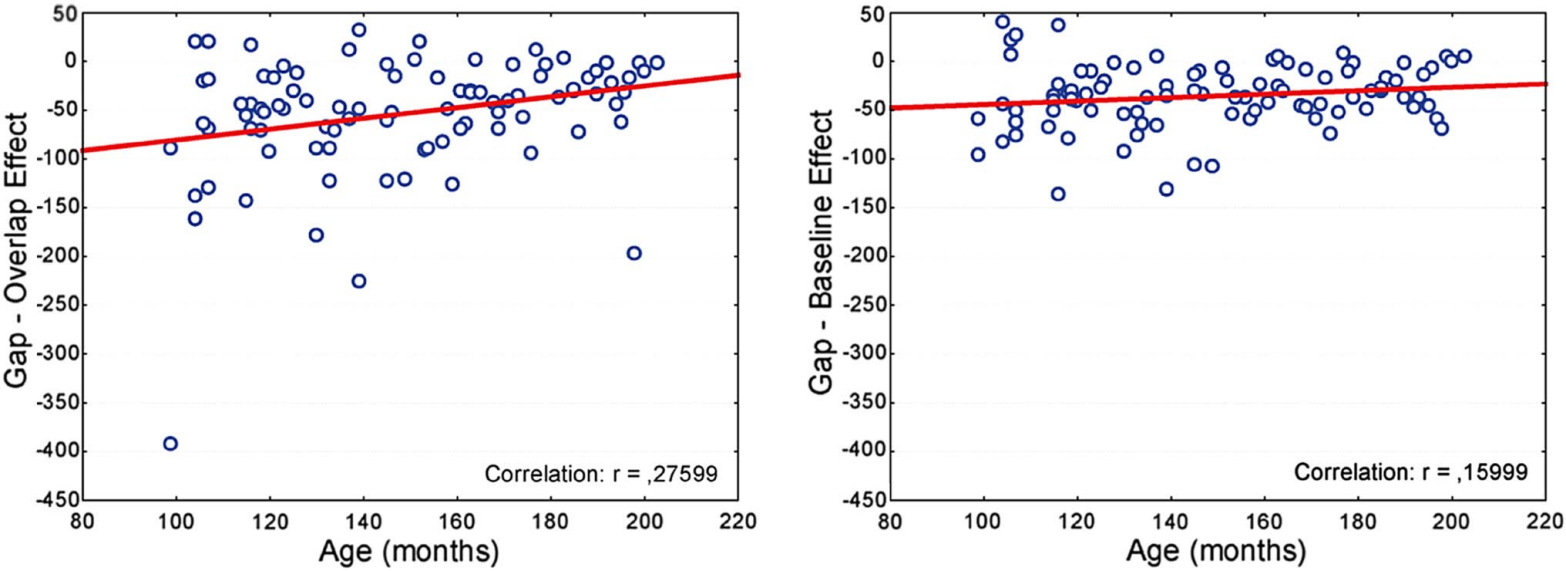
Result for the correlational analyses between age and both the gap-baseline and the gap-overlap effect

A Pearson correlation showed no significant correlation between age and the gap-baseline effect (*r* = 0.160; *p* = 0.13). A significant positive correlation was observed between age and the gap-overlap effect (*r* = 0.276; *p* < 0.01). Given that a stronger gap effect is indicated by a negative value, this analysis reveals that the gap-overlap effect decreases over age. See Fig. 2 for a graphical illustration of these two correlational analyses.

One might argue that the correlation for the gap-overlap effect is driven by one negative outlier in the 8-year-old group. Excluding this outlier still results in a significant correlation (*r* = 0.219; *p* ≤ 0.04), however.

Pearson and Filon’s test for overlapping correlations for dependent groups showed that these two correlations were not significantly different (two-tailed; *z* = −1.26; *p* = 0.21). This lack of a statistical difference is consistent with the idea that the baseline condition reflects an intermediate condition with respect to the amount of attentional disengagement.

In the gap condition, there is (almost) full disengagement, while there is little to no disengagement in the overlap condition. The baseline is then the intermediate condition; on the one hand, the amount of attentional disengagement in the baseline condition is not strong enough to observe a significant correlation between age and the difference between the gap and the baseline condition (in contrast to the difference between the gap and the overlap condition). On the other hand, there is already some disengagement in the baseline condition, resulting in a non-significant comparison between the two correlations. Our most important finding for these correlations is, therefore, that the effect of age is best reflected in the gap-overlap effect.

## Discussion

The aim of the present study was to investigate the ability to disengage visual attention in a group of children aged between 9 and 15. To this end, we employed the gap paradigm which is known to be a sensitive tool to study attentional disengagement. Besides the gap and the overlap conditions, we also added a baseline condition in which the fixation point was removed at the moment that the target appeared (i.e. a gap of zero ms). This way, we had two measures of the gap effect, namely the difference between the gap and the baseline condition and the difference between the gap and the overlap condition. This allowed us to reveal the appropriate task parameters to unravel possible developmental differences.

In line with numerous previous (adult) studies, we observed a clear gap effect for both measures of the gap effect (Saslow 1967) latencies of the saccades towards the target were shorter in the gap condition than in the overlap and baseline conditions. This decrease in saccade latencies in the gap condition is explained by the idea that attention is already disengaged when the peripheral target stimulus appears. This is in contrast to the overlap and the baseline condition, in which attention is still (partly) engaged to the initial fixation point when the peripheral stimulus appears. Furthermore, consistent with some earlier studies (e.g., Klein and Foerster 2001), there was a general effect of age in that saccade latencies were shorter for older children compared to the younger children in our sample.

Correlation analyses showed that the size of the gap effect became smaller with increasing age, but only for the difference between the gap and the overlap conditions. There was no significant correlation between age and the difference between the gap and the baseline conditions. This shows that there is indeed a gradual increase in the capacity to disengage visual attention with increasing age, in line with earlier observations by Klein (2001; Klein and Foerster 2001), but that this effect only becomes apparent when the gap and the overlap conditions are compared. This discrepancy with the studies that did not find such a correlation (Eenshuistra et al. 2007; Goepel et al. 2011) could be explained by a difference in statistical power. Both these studies tested a smaller group of participants compared to our study: the total number of subjects in both studies was less than the number of participants in one age group in our study. Furthermore, Goepel et al. (2011) also noted that the slope of their regression line was consistent with a decreasing gap effect with increasing age.

Given the fact that the frontal cortical regions mature throughout development (Fuster 1997), the observed developmental differences are consistent with the idea that the disengagement of attention is, at least for an important part, a frontal process (Dias and Bruce 1994). Subcortical areas that are known to play a role in the gap effect, such as the superior colliculus (Dorris and Munoz 1995), do not show such a development (Stampalija and Kostovic 1981; Yakovlev and Lecours 1967). The gap paradigm can, therefore, indeed be adopted as a marker for attentional control throughout the life span. The fact that the experiment can already be administered in infants (Cousijn et al. 2017; Hood and Atkinson 1993; Nakagawa and Sukigara 2013) makes it an attractive test to compare performance in a wide range of age groups. It should be noted, however, that developmental changes in the oculomotor system are profoundly different in the first years of development compared to those in later stages of development (Johnson 1990) and that our conclusions might, therefore, not generalize to early development (see e.g., Nakagawa and Sukigara 2013).

Our results further confirm the reasoning that the baseline condition is not a very valuable condition. In the baseline condition, the disengagement is not yet complete and it, therefore, reflects an intermediate condition compared to the overlap condition in which there is little to no disengagement. Further support for the overlap condition as a more informative condition comes from the observation that the test–retest correlation is better for the overlap-gap effect compared to the baseline-gap effect (Cousijn et al. 2017).

In sum, we show that the attentional system becomes more efficient with increasing age and that this is a gradual process. This observation is consistent with the idea that the gap effect is a useful measure to study frontal cortex development in healthy and clinical populations. Previous inconsistent results might be explained by a lack of statistical power in some of the studies. This type of basic knowledge about the gap effect is needed to reliably assess clinical groups.
